# Emergence of Rarely Reported Extensively Drug-Resistant *Salmonella* Enterica Serovar Paratyphi B among Patients in East China

**DOI:** 10.3390/antibiotics13060519

**Published:** 2024-06-02

**Authors:** Jiefu Peng, Jingchao Feng, Hong Ji, Xiaoxiao Kong, Jie Hong, Liguo Zhu, Huimin Qian

**Affiliations:** 1 NHC Key Laboratory of Enteric Pathogenic Microbiology, Jiangsu Provincial Center for Disease Control and Prevention, Nanjing 210009, China; pengjiefu@jscdc.cn (J.P.);; 2 School of Public Health, Xiamen University, Xiamen 361102, China; 3 Vanke School of Public Health, Tsinghua University, Beijing 100084, China

**Keywords:** *Salmonella* Paratyphi B, ST2814, XDR, ICE

## Abstract

Background: In recent years, global concern over increasing multidrug resistance (MDR) among various *Salmonella* serotypes has grown significantly. However, reports on MDR *Salmonella* Paratyphi B remain scarce, let alone the extensively drug-resistant (XDR) strains. Methods: In this retrospective study, we investigated the isolates of *Salmonella* Paratyphi B in Jiangsu Province over the past decade and carried out antimicrobial susceptibility tests, then the strains were sequenced and bioinformatics analyses were performed. Results: 27 *Salmonella* Paratyphi B strains were identified, of which the predominant STs were ST42 (11), ST86 (10), and ST2814 (5). Among these strains, we uncovered four concerning XDR *Salmonella* Paratyphi B ST2814 strains (4/5) which were previously unreported. These alarmingly resistant isolates showed resistance to all three major antibiotic classes for *Salmonella* treatment and even the last resort treatment tigecycline. Bioinformatics analysis revealed high similarity between the plasmids harbored by these XDR strains and diverse *Salmonella* serotypes and *Escherichia coli* from China and neighboring regions. Notably, these four plasmids carried the *ramAp* gene responsible for multiple antibiotic resistance by regulating the AcrAB-TolC pump, predominantly originating from China. Additionally, a distinct MDR ST42(1/11) strain with an ICE on chromosome was also identified. Furthermore, phylogenetic analysis of global ST42/ST2814 isolates highlighted the regional specificity of these strains, with Jiangsu isolates clustering together with domestic isolates and XDR ST2814 forming a distinct branch, suggesting adaptation to local antibiotic pressures. Conclusions: This research underscores the pressing need for closely monitoring the MDR/XDR *Salmonella* Paratyphi B, particularly the emerging ST2814 strains in Jiangsu Province, to effectively curb its spread and protect public health. Moreover, surveillance should be strengthened across different ecological niches and genera to track resistance genes and horizontal gene transfer elements under the concept of “ONE HEALTH”.

## 1. Introduction

*Salmonella* Typhi and Paratyphi are enteric bacteria that cause enteric fever and gastroenteritis. These infections pose a significant global health burden, with an estimated 11–21 million typhoid fever cases and 5 million paratyphoid fever cases occurring annually, resulting in 135,000–230,000 deaths worldwide [1]. While typhoid fever is preventable through vaccination, paratyphoid lacks an effective vaccine, making it a persistent public health concern in many developing regions [2,3].

*Salmonella* Paratyphi B can be categorized into two distinct biotypes based on dextro-tartrate (dTa) fermentation and slime wall formation: biotype *sensu stricto* (dTa-negative, slime wall-positive) and biotype Java (dTa-positive, slime wall-negative). Biotype Java strains primarily cause gastroenteritis and have been responsible for outbreaks of varying severity in Switzerland [4], the United States [5], and Spain [6]. Further, they also contribute to invasive disease, particularly among children, in travelers worldwide [7,8]. There has been an increase in reports of human infections with this biotype in recent years [9,10]. The geographical and source distribution of the strain is extensive, encompassing isolations from various sources such as amphibians [11], vegetables [12], and fish [13], with an increasing rate of isolation from poultry and poultry meat in Europe [14]. While most reports of MDR strains have focused on poultry and food sources [14,15], systematic investigations of human-infecting strains remain scarce.

First-line treatment of *Salmonella* infections relies on fluoroquinolone antibiotics, third-generation cephalosporins, and azithromycin [16]. Quinolone resistance arises through two mechanisms: chromosome-mediated quinolone resistance-determining region (QRDR) mutations, which alter drug target sites (DNA gyrase and topoisomerase IV), and plasmid-mediated quinolone resistance (PMQR), where genes carried by plasmids like *qnrA*, *qnrS*, and *aac(6′)-Ib-cr* confer resistance [17]. Resistance to third-generation cephalosporins predominantly involves the carriage of extended-spectrum β-lactamases (CTX, TEM, SHV types) [18]. Macrolide resistance mechanisms include specific resistance genes (*mphA*, *erm(42)*, *ermB*) and mutations in target genes or efflux pumps (*acrA*B:R717Q/L) [19,20]. Additionally, overexpression of multidrug efflux pumps (*acrA*B-*tolC*), regulated by genes like *ramAp* (the *ramA* variant located on plasmids), can also acquire a macrolide-resistant phenotype by enhancing antibiotic efflux [21,22]. Mobile genetic elements (MGEs) harboring drug-resistance genes can facilitate interspecies transmission. While previous research has focused on plasmids, chromosomally located MGEs have not been reported [23].

We have observed a concerning rise in *Salmonella* Paratyphi B infections within our province in recent years. However, not much was known about the genetic characteristics and drug resistance profiles of these strains. And, although Paratyphi B is a notifiable disease in China, few reports document its prevalence. The only reported strain from children with diarrhea was fully drug-sensitive [24]. Therefore, in this paper, we report the resistance profiles, mechanism of resistance, and evolution of *Salmonella* Paratyphi B strains in this province from 2013 to 2022.

## 2. Results

### 2.1. Salmonella Paratyphi B Isolates in Jiangsu

A total of 27 *Salmonella* Paratyphi B strains were double-confirmed by serum identification and whole genome sequencing analyses. Among these, 17 were identified as the Java biotype, while 10 belonged to the *sensu stricto* type. Multilocus sequence typing (MLST) analysis revealed four different sequence types (ST) within these strains: ST42 (11), ST86 (10), ST2814 (5), and ST135 (1). ST42, ST2814, and ST135 corresponded to the Java biotype, while ST86 represented *sensu stricto*. This suggests a potential association between specific STs and biotypes within *Salmonella* Paratyphi B.

### 2.2. Pan-Genome and Phylogenetic Analysis of Salmonella Paratyphi B Isolates Collected from Jiangsu

**Figure 1:**
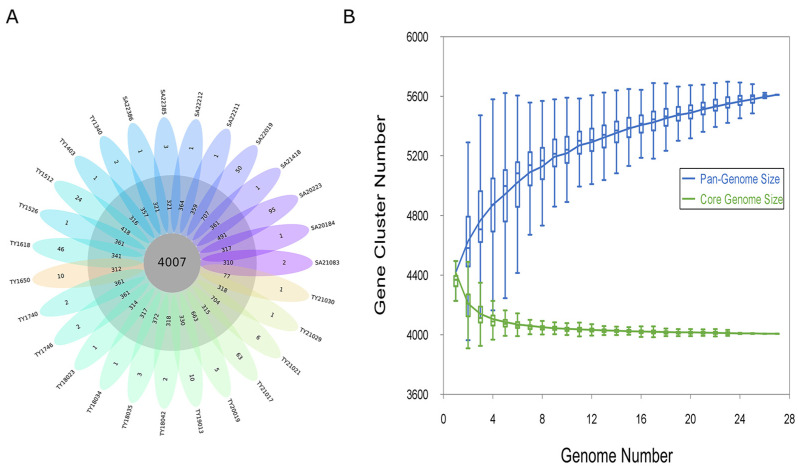
Pan-genome analysis of the *Salmonella* Paratyphi B genome. (**A**) Flower plot of 27 *Salmonella* Paratyphi B genomes showing the gene content of the core genome (center), the accessory genome (around the center), and strain-specific genes (petals). (**B**) Progressive curves for the core and pan-genome were estimated for all 27 genomes. Curves show the downward trend of the core gene families and the upward trend of the pan-gene families with additional genomes.

A pan-genomic analysis of 27 *Salmonella* Paratyphi B genomes revealed 5215 homologous gene families. In total, 76.8% (4007 genes) constituted the core genome, shared by all isolates. The accessory genome, encompassing 15.6% (814 genes), exhibited a presence in at least one genome, while the remaining 7.6% (394 genes) were unique to individual strains, highlighting strain-specific genomic diversity (Figure 1A). Further, the Heaps’ law estimate supported a closed pangenome (α = 1.37) (Figure 1B)

**Figure 2:**
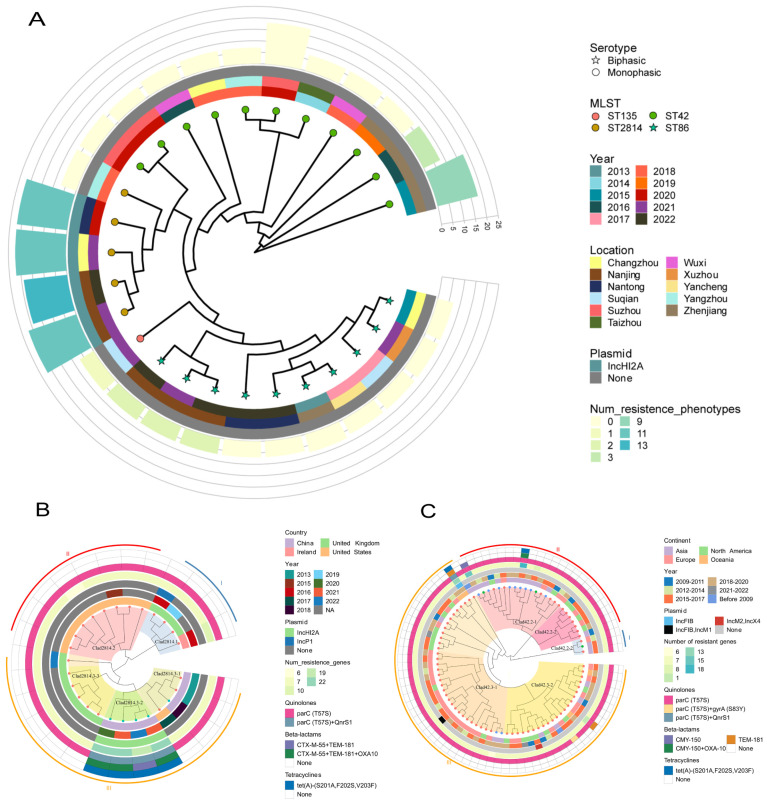
Phylogenetic trees reconstructed from SNP data using the maximum likelihood method. (**A**) Phylogenetic tree of 27 strains of Paratyphi B in Jiangsu province, ○ and ☆ on the node represent monophasic and biphasic separately; the colors represent different MLST; and, from the inner circle to the outer circle—the first circle represents the year of the isolation of the strains, the second represents the region where the strains were isolated, the third represents the presence or absence of the plasmids, and the height and color of the fourth circle represent the number of resistance genes and the resistance phenotype, respectively. (**B**) Phylogenetic tree of 26 ST2814 strains worldwide; the colors on the node represent the strains isolated in the province (green) and the downloaded strains (red); and, from the inner circle to the outer circle—the first circle adjacent represents the country in which the strain was isolated, the second represents the year of isolation, the third represents the year of plasmid replicon isolation, the fourth represents the number of resistance genes, and the fifth, sixth, and seventh circles represent the carriage of quinolone, beta-lactam, and tetracycline resistance genes, respectively. (**C**) Phylogenetic tree of 85 ST42 strains from all over the world, and the colors on the node represent the isolates from this province (blue) and the downloaded strains from other parts of China (green) and the downloaded non-Chinese strains (red). The first circle indicates the continent where the strain was isolated, and the other circles represent the same meanings as in Figure 2B.

Phylogenetic analysis of 27 Jiangsu *Salmonella* Paratyphi B isolates based on 33,127 core SNPs revealed distinct evolutionary branches (Figure 2A). ST2814 java isolates clustered tightly (4–42 SNPs) and had a smaller sub-cluster (4–10 SNPs) with strains collected from this province from 2020 to 2022 (TY20019, TY21021, TY21030, and SA22019). Conversely, ST86 *sensu stricto* isolates displayed wider diversity (1–498 SNPs) and longer-term presence in the province (across the whole decade).

### 2.3. Phylogenetic Analysis of the Global Sequences of ST2814 and ST42

Whole-genome SNP analysis of our five ST2814 strains alongside 21 global isolates from the United Kingdom (*n* = 9), the United States (*n* = 9), Ireland (*n* = 1), and China (*n* = 2) revealed 648 SNPs and three distinct clades with pronounced regional clustering. Notably, most of the USA (clad2814.1) and UK strains (clad2814.2 and clad2814.3-3) formed separate branches, while most Chinese strains diverged significantly. Within our province, all ST2814 strains belonged to the diverse clad2814.3, with one 2018 isolate clustered in the clad2814.3-1 branch along with one 2017 US strain and two 2013 strains from the other regions of China, while four 2020–2022 isolates formed a unique sub-lineage (clad2814.3-2) which highlights the emergence of a potentially localized subpopulation (Figure 2B).

Genome-wide SNP analysis of 11 ST42 strains from our province alongside 74 global strains unveiled 17,168 informative loci. The resulting phylogeny revealed two major branches, each exhibiting a broader temporal and geographical distribution. Furthermore, nine Jiangsu strains resided within clad42.2-1 alongside diverse isolates from Europe, North America, and Asia. And four of them formed a distinct sub-lineage, suggesting potential local evolution. The remaining two Jiangsu strains clustered within clad42.3-1, demonstrating closer relationships with European strains. This diverse distribution within the global ST42 population highlights the complex interplay between regional and temporal dynamics shaping strain evolution (Figure 2C).

### 2.4. Antibiotic Resistance Profile and Associated Resistance Determinants

The antibiotic resistance profiles of these strains were determined by testing their susceptibility to a panel of major antibiotic classes, including beta-lactams, tetracyclines, quinolones, macrolides, aminoglycosides, sulfonamides, trimethoprim, amphenicols, and polymyxins. Most strains (17/27) were almost pan-susceptible, but 10 displayed six unique resistance profiles (Table S1). Five strains were multidrug resistant, four of which are XDR (TY20019, TY21021, TY21030, and SA22019) (clad2814.3-3), and they were resistant to every class except polymyxins (Figure 2B). Another MDR strain (clad42.2-1) showed susceptibility to quinolones, macrolides, and polymyxins but resisted all other classes; the remaining five strains (three ST86 strains, one ST42 strain, and one ST135 strain) showed resistance to one to four antibiotics, respectively (Figure 2C). Interestingly, XDR and MDR strains were exclusively associated with Java biotypes, while other resistant strains exhibited both Java (2/5) and *sensu stricto* biotypes (3/5).

**Figure 3:**
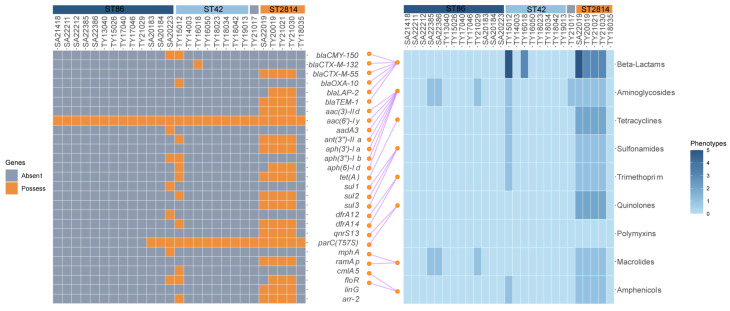
Resistance genes, resistance phenotypes, and the correspondence between them of the 27 strains: The left side shows the resistance genes of strains, the yellow square represents the existence of the resistance gene, while the grey square indicates its absence; on the right side are the antibiotics susceptibility testing (AST) results of the eight major classes of antibiotics, and the color shades represent the number of antibiotics resistant to this antibiotic class; the connecting lines in the middle represent the corresponding relationship between resistance genes and resistance phenotypes (from the literature and the CARD database); the corresponding ASTs for the rifamycins and lincosamides, which corresponded to the arr-2 and the linG, were not carried out, and therefore there were no connecting lines between them.

A total of 36 resistance genes were identified across the 27 strains, falling into ten categories based on their antibiotic target. Most strains exhibited good concordance between their resistance genotypes and phenotypes (Figure 3). XDR strains harbored eight categories with 20–23 genes, while MDR strains carried seven classes with 18 genes. Other resistant strains carried only six to eight genes. However, ST2814 and ST42 isolates from other areas of the world harbored significantly fewer resistance genes, averaging only seven per strain with a maximum of 13, this stood in stark contrast to our regional finding of XDR and MDR strains exceeding 18 genes (Figure 2B,C). Additionally, the presence of *Salmonella* genomic islands (SGI1, SGI1-K, and SGI2) was not observed across all tested strains.

### 2.5. Quinolone, Third-Generation Cephalosporin, and Tetracycline Resistance Mechanism

We screened all 27 strains from our study for the QRDR mutation and the PMQR gene. Analysis revealed that only the Java strains (ST42 and ST2814) harbored a single amino acid substitution (T57S) in the *parC* gene, which was confirmed by PCR test and sanger sequencing. The same results were obtained when we analyzed other global ST42 and ST2814. While only one PMQR gene was detected, the four XDR strains (15%) which showed high-level resistance to ciprofloxacin (2 mg/L) all harbored the *qnrS13* gene.

Six strains (22%) harbored β-lactamase genes, conferring resistance to third-generation cephalosporins (cefotaxime and ceftazidime). These genes included extended-spectrum β-lactamase (ESBL) genes like *bla*_CTX-M-55_ (*n* = 4) and *bla*_CTX-M-132_ (*n* = 1), along with a diverse array of other β-lactamases: *bla*_TEM-1_ (*n* = 4), *bla*_OXA-10_ (*n* = 1), *bla*_CMY-150_ (*n* = 1), and *bla*_Lap-2_ (*n* = 3). Further, one MDR strain (TY1512) harbored both *bla*_CMY-150_ and *bla*_OXA-10_, while another XDR strain (SA22019) carried two copies of *bla*_CTX-M-55_ and *bla*_TEM-1_. These two strains exhibited additional resistance to cefoxitin (MIC = 64 mg/L, 32 mg/L) and ampicillin/sulbactam (MIC = 32/16 mg/L).

Five strains (19%) possessed the *tet(A)* gene, all harboring double-shift mutations (S201A, F202S, V203F). These strains exhibited tetracycline resistance (MIC > 16 mg/L), and four harbored plasmid-borne *tet(A)*, additionally demonstrating tigecycline resistance (two MIC= 1 mg/L and two MIC = 2 mg/L).

### 2.6. Transferability Test of Plasmids of XDR Strains

**Figure 4:**
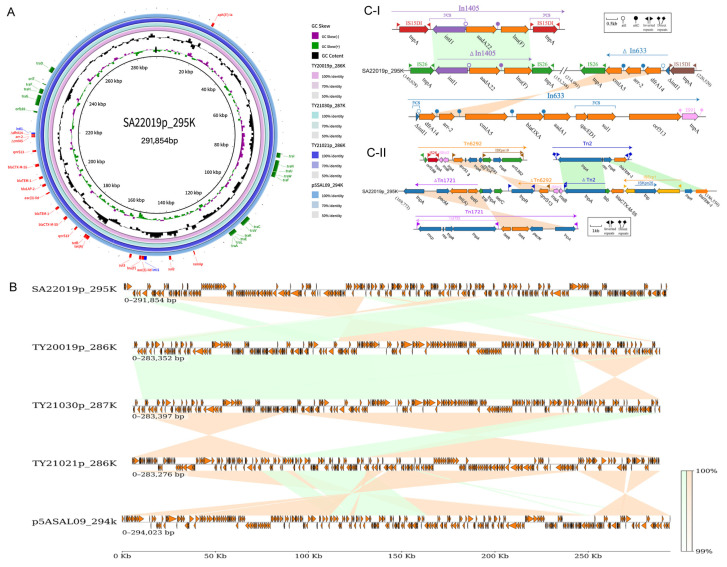
Comparative genome and linear analysis of XDR plasmids. (**A**) Comparative genome map of five plasmids with SA20019p_295K in the center of the map, and TY20019p_286K, TY_21030p_287K, TY21021p_286K, and p5ASAL09_294k as the circles from the inner to the outer; the annotated genes on the outer-most layers are the resistance genes (red), integrase genes (blue), and conjugation-related genes (green) of SA22019p_295K; the shades of the circles represent the sequence similarity between the four plasmids and SA20019p_295K, respectively. (**B**) Full-length linear analysis of the five plasmids, with green representing the forward-sequence similarity, yellow representing the reverse-sequence similarity, and the color shade representing the sequence similarity percentage. (**C**) Linear comparison of the SA20019p_295K XDR regions with In1405, In633 (I), Tn6292, truncated Tn1721, and truncated Tn2 (II), with green representing the forward-sequence similarity, yellow representing the reverse-sequence similarity, and the color shade representing the sequence similarity percentage.

Transferability analysis of plasmids in the four XDR strains revealed potential transferability for all four, with SA22019p_295 K exhibiting characteristics of a potential conjugative plasmid (harboring oriT, T4ss genes cluster, T4cp, and Relaxase) (Figure 4A). This potential was confirmed through conjugation experiments, where the recipient strain EC600 gained resistance to 10 additional antibiotics upon acquiring the plasmid, and MICs against other three antibiotics increased (Table S1). Also, full coverage was achieved by mapping the clean data of the transconjugant against SA22019p_295K sequence, which verified the success of the conjugation experiments.

### 2.7. Genetic Localization of the MDR Region on Plasmids

Plasmids of four XDR strains (SA22019, TY20019, TY21021, and TY21030) were identified and verified by PCR as IncHI2A; screening against PLSDB showed high similarity to plasmids from diverse *Salmonella* serotypes, with the closest match (99.2% coverage, 100% identity) being p5ASAL09_294k (CP090142.1) from a *Salmonella* Goldcoast isolate in Taiwan Province, China. Comparative genomic analysis confirmed high similarity among the five plasmids with average nucleotide identity (ANSI) ranging from 99.79% to 99.97% (Figure 4A), while linear analysis revealed extensive rearrangements, particularly within the multidrug resistance region (Figure 4B). Resistance gene analysis of SA20019p_295K identified 17 genes (including two copies of *qnrS13*, *bla*_TEM-1_, and *bla*_CTX-M-55_) conferring resistance to β-lactam, quinolone, sulfonamide, tetracycline, aminoglycoside, rifamycin, lincosamide, and multidrug classes (Figure 4A). The remaining three plasmids harbored an additional *floR* gene for amphenicol class resistance, alongside the 14 shared single-copy genes.

Resistance genes in SA20019p_295K were predominantly located between 14 kb and 22 kb, encompassing two clusters of ISs/transposons (16.9–19 kb and 19.5–22.1 kb) and two integrons (14.9–15.3 kb and 21.4–22.1 kb) with the second embed in the last ISs/transposons cluster. Linear analysis revealed both integrons as transferable structures flanked by insertion sequences. The first integron, resembling In1405 but with flanking IS26 instead of IS15D1, harbored *int1*-Ant(3″)-Ia-*lnu(F)* units. The second integron comprised the IS26-*ΔcmlA5-arr-2-dfrA14-ΔintI*-IS15DI unit, where *ΔcmlA5-arr-2-dfrA14-ΔintI* originated from truncated In633. (Figure 4(C-I)) A 476 bp residue deletion in *cmlA5* rendered this pseudogene non-functional. Moreover, the first ISs/transposons cluster harbored *tet(A)*, *qnrS13*, *bla*_CTX-M-55_, and *bla*_TEM-1_ genes derived from truncated Tn6292, Tn1721, and Tn2, respectively; the first two Tns underwent a complete inversion within SA20019p_295K, while Tn2 was split, with *tnpA* inserted before *bla*_CTX-M-55_ and the *tnpR*-*bla*_TEM-1_ segment moved to the end of the cluster (Figure 4(C-II)). The second ISs/transposons cluster harbored another copy of *bla*_TEM-1_, *bla*_CTX-M-55_, and *qnrS13* alongside two drug-resistant genes within the second integron.

### 2.8. Analysis of Multidrug-Resistance ramAp-Related Genes’ Expression and Genetic Environment

**Figure 5:**
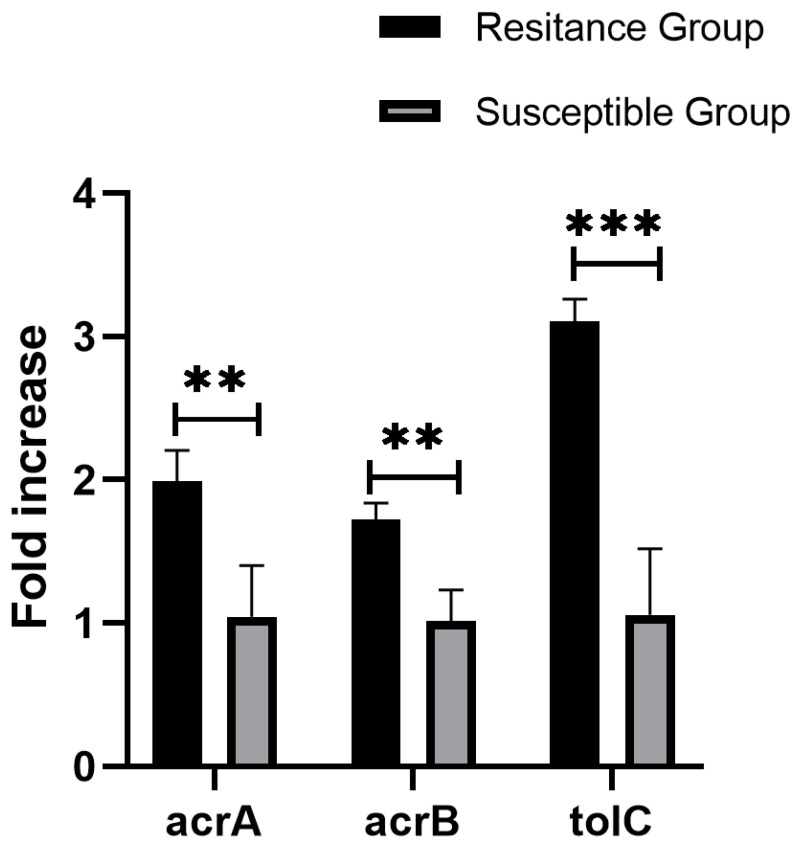
Average expression levels of *acrA*, *acrB*, and *tolC* genes in *ramAp* harbored strains, values on the *y*-axis are relative expression levels (fold increase) normalized against levels of the endogenous reference *rpoB*. The data correspond to the mean values of three biological replications. Error bars correspond to the standard deviation. Asterisks indicate statistically significant differences (** indicate *p* < 0.01, *** indicate *p* < 0.001) in student *t*-tests.

**Figure 6:**
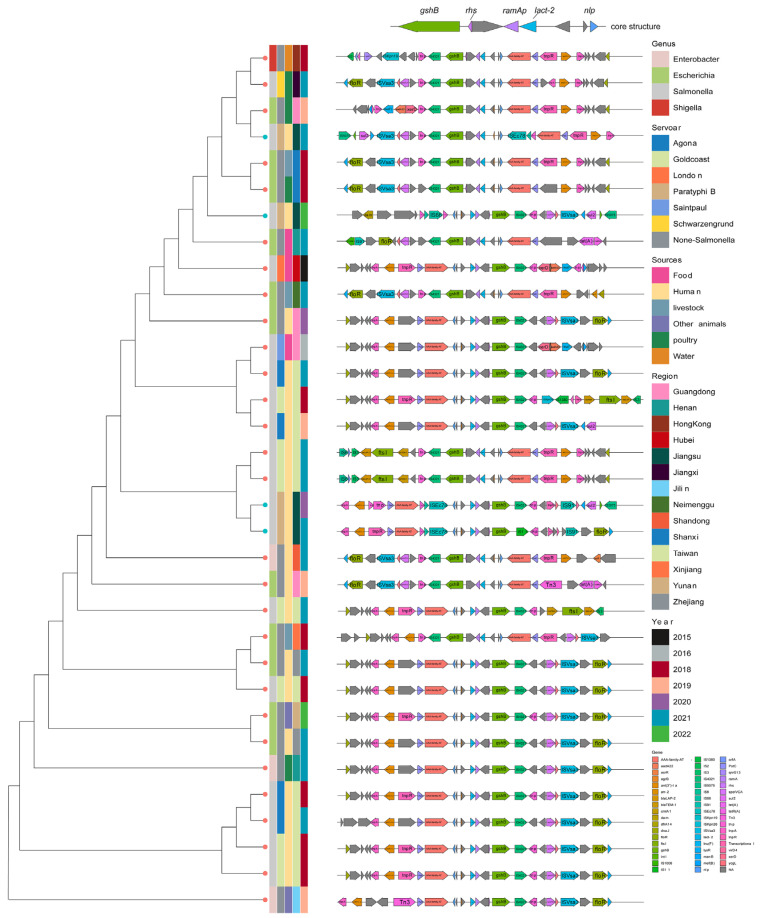
Comprehensive information and evolution relationship among 33 *ramAp*-bearing plasmids. Plasmids were colored according to genus, serotype (if the strain belongs to *Salmonella* Genus), collection source, collection provinces, collection year, and genetic context of *ramAp* from left to right.

The presence of *ramAp* in plasmids significantly enhances multidrug resistance by activating the AcrAB-TolC efflux pump in various bacteria. All XDR strains harbored plasmids containing *ramAp*, its upstream regulatory region, and the truncated ISEcp1. Also, their downstream genes (*acrA*, *acrB*, *tolC*) exhibited 1.6–3-fold higher expression compared to sensitive strains (*p* < 0.01, *t*-test) (Figure 5). NCBI database analysis revealed 53 other global plasmids harboring identical *ramAp* genes and upstream promoters, mainly from Asia (86.79%), North America (7.55%), and Australia (1.89%). Additionally, 25 were collected from human specimens, while others originated from food, poultry, and livestock sources. *Salmonella* (*n* = 25) and *Escherichia coli* (*n* = 22) dominated the distribution, with *Salmonella*’s major serotypes being Goldcoast (*n* = 9) and Agona (*n* = 8). Geographically, 25 *Salmonella* strains originated from China, with 10 from Taiwan and 7 from other provinces. Plasmid types included IncHI2 (*n* = 40), IncHI2-IncN fusions (*n* = 9), InC (*n* = 3), IncFIA(HI1)-IncR fusions (*n* = 1). Transferability prediction indicated 37 of the 53 plasmids as transferable, including two potential conjugative plasmids. Further analyzing 33 plasmids from 29 China-derived strains and our four XDR strains, we constructed a phylogenetic tree. This analysis revealed that a plasmid from a Taiwan Province patient’s *Salmonella* Goldcoast strain closely resembled TY20019p_286K and TY21030p, while SA22019p_295K and TY21021p_286k aligned with *E. coli* plasmids from poultry sources in Shanxi and Guangdong, respectively (Figure 6). Examining the *ramAp* gene surroundings revealed a conserved structure: *tnpA-pstC*-(AAA-family-ATPase)-*nlp-argB*-(*lact-2*)-*ramAp*-*gshB*-IS4321, additionally, most (27/33) harbored the *tnpR*-Ant(3″)-Ia-*tnpA* transposon with an aminoglycoside resistance gene at the 5′ end.

### 2.9. Genetic Localization of the MDR Region on Chromosomes

**Figure 7:**
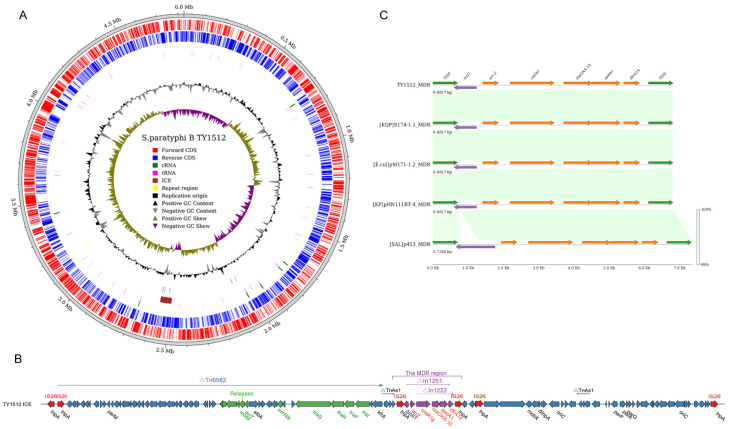
ICESml1512 structure and MDR region analysis. (**A**) Structure of TY1512 chromosome and ICESml1512 localization; (**B**) fundamental structure of ICESml1512, with green representing integrative, conjugative related genes and purple representing drug resistance genes; (**C**) linear analysis of the ICESml1512 MDR region with MDR regions from the *Klebsiella quasipneumoniae* (S174-1.1), *Escherichia coli* (pM171-1.2), *Klebsiella pneumoniae* (pHN11RT-4), and *Salmonella Typhimurium* (p453) plasmids, with the color shades representing sequence similarity.

Analysis using ICEfinder revealed a putative ICE structure (ICESml1512) on the chromosome of the multidrug-resistant strain TY1512 (Figure 7A). This 70,174 bp structure, unique upon BLAST comparison, had an average GC content of 49.3% and an upstream site-specific DNA recombinase. Its genomic composition included an integron with five tandem resistance genes, metabolism-related genes, and ABC transporter system-related genes. It also possessed the *oriT*, recombinase, relaxase, and T4SS gene clusters crucial for integration and conjugation (Figure 7B). Comparative analysis showed the first 36,000 bp sharing 94% coverage and 99.9% identity with ICE (Tn6582) from *Klebsiella pneumoniae*. Following this segment was the MDR region, flanked by two IS26 insertion sequences and harboring a novel class I integron structure, *intI-arr-2*-*cmlA5*-*bla*_OXA-10_-*aadA1*-*dfrA14*. Derived from truncated In1251 and In1222, these five genes conferred resistance to rifamycin, acylated, β-lactam, aminoglycoside, and methotrexate antibiotics, respectively. This MDR region was identical to those found in plasmids from *Escherichia coli* (pM171-1.2, CP101668), *Klebsiella quasipneumoniae* (S174-1.1, CP063875.1), and *Klebsiella pneumoniae* (pHN11RT-4_MDR, CP125892), which were collected from wild animals (the first two) and the environment, respectively in Guangdong, China. In addition, the only discernible dissimilarity between this plasmid and a *Salmonella* plasmid (p453, CP060856.1) obtained from a patient in Zhejiang, China, was observed within the integrase region. These findings suggest the presence of a potentially mobile MDR element capable of horizontal transfer within *Enterobacterales* via integration and excision mediated by ICE or plasmids (Figure 7C).

## 3. Discussion

The alarming global rise of multidrug resistance (MDR) in *Salmonella* has thrust this pathogen into the spotlight. Serotypes like Typhi, Typhimurium, and Indiana increasingly contribute to major human epidemics with their escalating resistance profiles [25,26,27]. To address this pressing concern, we conducted the first drug resistance analysis of *Salmonella* Paratyphi B in China using a 10-year retrospective study of clinical isolates from Jiangsu Province. Our findings not only shed light on the potential drivers of MDR emergence in this serotype but also reveal the first-ever human-infected ST2814 XDR Java biotype strains persisting in the region for almost three years. This unprecedented discovery signifies a potential turning point in the antibiotic resistance of *Salmonella* Paratyphi B and warrants vigilant monitoring.

*Salmonella* Paratyphi B become the dominant cause of typhoid and paratyphoid in the region in recent years, deviating from both national and international trends where Typhi and Paratyphi A typically prevail [28,29]. While ST43 and ST149 dominate *Salmonella* Paratyphi B isolates in Europe [11], Jiangsu’s prevalent types are ST42/ST2814 (Java biotype) and ST86 (*sensu stricto* type). Intriguingly, ST2814 is rarely encountered in global databases. Analyzing the global phylogenetic tree for ST42/ST2814 revealed that Jiangsu isolates clustered with domestic strains; this distinct clustering compared to foreign strains suggests local or national endemicity of the prevalent branches. Furthermore, while the majority of global ST42 and ST2814 strains exhibit potential highly antibiotic susceptibility, our provincial strains, particularly the XDR ST2814, diverged into a separate branch, potentially indicating their heightened adaptation to antibiotic selection pressures.

Among the diverse array of ESBL enzymes in this study, *bla*_CTX-M-55_ held the highest prevalence, reflecting its dominance among human- and animal-derived *Salmonella* in China [30,31]. While *bla*_CTX-M-55_ typically resides within the “ISEcp1-*bla*_CTX-M-55_” element, XDR strains in this study exhibited a unique flanking chimeric form structure: “ΔISEcp1-ISKpn26-ΔISEcp1-*bla*_CTX-M-55_” (Figure 4C), which may enhanced conjugative transfer potential. Moreover, *bla*_CTX-M-55_ is mostly derived from the IncHI2, IncI2, and IncF replicon type plasmids in all kinds of *Salmonella* collections [32,33], with the highest carryover in the IncHI2 plasmid, which can mediate the transfer of resistance genes between genera, it was also frequently associated with other resistance genes such as *bla*_TEM-1_, *bla*_TEM-141_, and qnrS1 [34], partially intersecting with all four XDR strains in this study. Additionally, coexistence and multiple copies of enzymes will further compromise the inhibitory effect of sulbactam.

Four XDR strains resistant to nalidixic acid and ciprofloxacin harbored only parC mutations (T57S) and qnrS13 genes. However, the mutation at codon 57 of parC may be a potential compensatory mutation according to previous reports, which does not reduce the susceptibility of the strain to fluoroquinolone antibiotics [35]. Meanwhile, qnrS only caused a slight increase in the level of fluoroquinolone resistance especially when their expression was increased [36,37]. Therefore, neither of these two factors is the root cause of drug resistance. Additionally, XDR strains possessed the ramAp gene, which enhances AcrAB-TolC pump activity, leading to a 2–4-fold increase in nalidixic acid and ciprofloxacin resistance [22]. This suggests that the observed fluoroquinolone resistance in XDR bacteria likely arises from the expression of the ramAp gene.

Tigecycline, a last resort antibiotic against multidrug-resistant bacteria, faced some resistance from *Salmonella* Paratyphi B in this study. Double mutations (S201A, F202S, V203F) in the Tet(A) protein’s C3 interdomain loop region were previously reported in other *Salmonella* [38], causing low-level resistance due to altered substrate specificity. In addition, strains harboring both these *tet(A)* mutations and the *ramAp* gene exhibited resistance to both tetracycline and tigecycline. Both genes augment the activity of different efflux pumps, playing a pivotal role in *Salmonella*’s tigecycline resistance. This combination of efflux mechanisms suggests a potential synergistic effect, confirming observations in Klebsiella pneumoniae [39].

A plasmid-borne transcriptional regulator, *ramAp*, emerged as a potential culprit for the resistance of azithromycin, chloramphenicol, and ciprofloxacin of the XDR isolates in this study. This gene, highly similar to Klebsiella quasipneumoniae’s chromosomal *ramA* [22], significantly boosted the expression of AcrAB-tolC efflux pump genes, which can result in the elevated MIC of diverse antibiotics including chloramphenicol, azithromycin, tigecycline, tetracycline, nalidixic acid, ciprofloxacin, and sulfamethoxazole [40]. This elevation was also confirmed by conjugation experiments in our study. Moreover, *ramAp* resides on plasmids exhibiting high interspecies conservation and widespread carriage across diverse hosts of the *Enterobacteriaceae* family. Through comprehensive global plasmid search and comparison, plasmids carrying the *ramAp* gene have been found to be of diverse types, most of which are highly similar to the plasmid replicon in this study and belong to the same IncHI2 type. These plasmids constitute a distinct evolutionary lineage predominantly found in Asia and exhibit a diverse range of host reservoirs, including animals, the environment, food sources, and patients. Our Jiangsu isolates shared high similarity with patient and poultry strains from other regions, suggesting the potential transferability of this plasmid and its associated efflux-mediated multidrug resistance across different ecological niches. Moreover, the *ramAp* gene was relatively well-conserved, and was typically situated within a core gene cluster flanked by the transfer elements *tnpA* and IS4321. This structure may facilitate *ramAp* exchange between different plasmids or chromosomes, ensuring its correct expression and function. This raises concerns about its potential for environmental adaptation, pathogenic impact, and the further spread of multidrug resistance. Therefore, close monitoring of *ramAp* carriage and dissemination is crucial for the effective management of this emerging threat.

While the IncHI2A plasmid is well-known for its MDR potential in diverse *Salmonella* strains [41], this study reveals its crucial role in *Salmonella* Paratyphi B XDR isolates. All four XDR strains harbored this plasmid, which contained resistance genes embedded within complex integrons and transposons, driving the MDR and even the XDR phenotypes. This finding contrasts with previous reports that attributed *Salmonella* Paratyphi B multidrug resistance to *Salmonella* genomic island 1 (SGI-1) or chromosomal localized class 1 integrons carrying the *pse-1* or *aadA2* gene cassette [42,43]. In addition, high sequence similarity and extensive inverted rearrangements within the plasmids suggest frequent recombination events between resistance gene fragments across these XDR strains. Moreover, the conjugative plasmid highlighted its ability to exacerbate resistance spread within *Salmonella* Paratyphi B populations in this region. Notably, similar plasmids have been identified in *Salmonella* and *E. coli* isolates from humans and poultry in China and neighboring countries (Supplementary Table S2), suggesting this resistance plasmid’s wider circulation and potential interspecies transmission, which necessitates further monitoring and research.

Our study identified an MDR strain (TY1512) harboring an integrative and conjugative element (ICE). Unlike plasmids, ICEs can propagate vertically through chromosomal integration and horizontally through excision like conjugative plasmids. This ICE in TY1512, carrying genes for resistance against five antibiotics classes, bears a remarkable resemblance to MDR regions identified in plasmids from diverse environmental, human, and animal sources across China. This implies efficient transfer capability, potentially including chromosome-to-plasmid or plasmid-to-plasmid jumps facilitated by the embedded IS26 element. Furthermore, the ICESml1512 encoded a site-specific recombinase (intI), a relaxase (Tral), and a type VI secretion system, enabling interchromosomal transfer of resistance and virulence factors via conjugative transfer and site-specific recombination. Limited prior research has been conducted on drug resistance-associated ICE in *Salmonella* [44], the emergence of such MDR ICE underscores the urgent need for enhanced surveillance and research on ICE dissemination and persistence in *Salmonella* and other genera of the *Enterobacteriaceae* across diverse ecological niches.

## 4. Materials and Methods

### 4.1. Bacterial Strain Collection and Identification

The Jiangsu Center for Disease Control and Prevention (JCDC) contributed to the National Pathogenic Bacteria Identification Network (NPIN) Surveillance and Typhoid/Paratyphoid Surveillance Programs by acquiring isolates from human specimens indicative of bacterial infection. All strains utilized in this study, encompassing the period 2013 to 2022, were subjected to identification via matrix-assisted laser desorption ionization time-of-flight mass spectrometry (MALDI-TOF-MS) (Bruker, Bremen, Germany), followed by confirmation using slide agglutination against the White Kauffmann–Le Minor scheme (serum kit from Statens Serum Institute, Copenhagen, Denmark).

### 4.2. Antimicrobial Susceptibility Testing

The Minimum Inhibitory Concentrations (MICs) of 21 antibiotics against *Salmonella* Paratyphi B strains were determined using a commercially available Gram-negative bacterial drug susceptibility assay plate with a Phoenix system (BD Biotechnology Co., Ltd., Baltimore, MD, USA). The specific types of antibiotics were shown in Table S1. *Escherichia coli* ATCC25922 served as the quality control strain, and the results, except tetracycline, were interpreted according to the Clinical and Laboratory Standards Institute (CLSI) guidelines outlined in document CLSI-M100-S3T2 [45]. The tetracycline results were interpreted according to EUCAST breakpoint table V14.0 since CLSI had no applicable standard [46]. MDR and XDR isolates were signed using typhoidal *Salmonella*’s criteria [47].

### 4.3. Whole Genome Sequencing, Assembly, Typing, and Plasmid Replicon Identification

The genomic DNA of positive isolates was extracted using the FastPure Bacteria DNA Isolation Mini Kit (Vazyme Biotechnology Co., Ltd. Nanjing, China) following overnight culture. Subsequently, DNA libraries were constructed and sequenced on an Illumina Novaseq platform, yielding paired-end reads of 150 bp. Finally, MDR and XDR strains underwent additional long-read sequencing via Oxford Nanopore according to the manufacturer’s protocol. The raw reads from all strains were processed through CLC Workbench for trimming and assembly. This platform also facilitated the generation of five complete genomes for the MDR and XDR isolates by employing a long-read assembly and short-read polish approach.

MLST for all strains was performed using EnteroBase [48]. Additionally, RhierBAPS [49] combined with SISTR [50] was employed for biotyping analysis. Plasmid replicons were identified using PlasmidFinder [51] and further determined by PCR [52].

### 4.4. Pan-Genome Analysis

OrthoFinder2 was used to obtain Orthologous groups of protein families of the pangenome with the DIAMOND method [53]. The results were used to extract the pangenome (total of all genes found across strains), core genome (genes shared among all strains), accessory genome (genes shared among more than one strain, but not in all), and strain-specific genes (genes found only in one strain). The open pangenome was determined with Heaps’ law (N = kn^−α^), in which α < 1 indicates an open pangenome.

### 4.5. Phylogenetic Analysis

Three SNP-based phylogenetic trees were constructed with RAxML followed by SNP calling and recombination removal with Snippy and Gubbins [54,55,56]; the first tree utilized sequences from strains obtained in this study. The second and third trees employed sequences from 11 ST42 and 5 ST2814 strains, alongside 74 representative ST42 strains from each continent and all 21 available ST2814 strains, respectively. (sourced from EnteroBase).

### 4.6. Analysis of Resistance Genes and Islands

*Salmonella* genomic island (SGI) sequences were primarily identified using MyDbFinder with reference sequences SGI1 (AF261825), SGI1-K (AY463797), and SGI2 (AY963803). A 60% coverage and identity threshold was employed to confirm SGI presence. Assembled genomes were analyzed using CLC with thresholds of 90% identity and 80% coverage to identify AMR genes and point mutations. The quinolone resistance-determining regions (QRDRs) were further validated using PCR and sanger sequencing [57].

### 4.7. MDR-Harboring Plasmids and ICE Analysis

Four complete plasmid sequences obtained from the hybrid assembly were subjected to comparative genomic analysis using Brig [58] and pyGenomeViz [59]. Open reading frames were initially predicted with Prodigal [60], followed by Bakta annotation [61], with further refinement and correction utilizing BLASTN/BLASTP [62] against the RefSeq database [63]. PLSDB facilitated the search for closely related plasmids [64].

Full-length chromosomes from the hybrid assembly were analyzed using ICEberg for ICE detection [65], followed by gene cluster analysis and linear comparison of predicted ICE structures. The MobileElementFinder [66], ISfinder [67], Transposon Registry [68], oriTFinder [69], and INTEGRALL [70] websites were used for comprehensive identification and annotation of all mobile elements.

### 4.8. Transferability Analysis and Conjugation Experiment

Conjugative elements were identified by oriTFinder; plasmids contain four necessary conjugative elements including the origin region of transfer (oriT), type VI system (T4SS) gene cluster, type VI coupling protein (T4CP) gene, and relaxase gene can be seen as potential conjugative plasmids [71,72]. Conjugation experiments were performed using strains containing potential conjugative plasmids as the donor and *E. coli* EC600 (rifampin-resistant) as the recipient. Both strains were grown to log phase (OD~0.6) in LB broth at 37 °C. Amounts of 400 μL of donor and 200ul of recipient cultures were mixed and incubated for 18 h at 37 °C. The cultures were then diluted 200-fold and spread on LB agar containing rifampicin (2000 μg/mL) and cefotaxime (4 μg/mL). Positive transconjugants were identified by mass spectrometry, antimicrobial susceptibility testing, and whole-genome sequencing.

### 4.9. Analysis of ramAp-Related Genes’ Expression and Genetic Localization

The presence of *ramAp* and genes within the four MDR plasmids was confirmed by BLASTN. Relative expression levels of *ramAp*-linked genes (*acrA*, *acrB*, and *tolC*) were measured using Hong et al.’s method [22], involving RNA extraction with an RNeasy mini kit (Qiagen), quality assessment using a NanoDrop One spectrophotometer (Thermo), qPCR on an ABI Quantstudiotm 7 Pro real-time PCR system using a Vazyme real-time RT-PCR kit, and normalization to the endogenous reference gene *rpoB*. Fold changes (2^−ΔΔCT^) were used to reveal expression differences between four *ramAp*-positive and four *ramAp*-negative strains, with statistical significance assessed via Student’s *t*-test by using GraphPad Prism 9.2 software. The primer (Table 1) concentrations equaled 200 nM and melt curve analysis ensured that only a single PCR product was amplified. The PCR mixture was reverse transcribed to 50 °C for 15 min, denatured to 95 °C for 2.5 min, followed by 40 cycles of 95 °C for 15 s, 55 °C for 20 s, and 72 °C for 20 s. The melt curve analysis was carried out immediately with ABI default procedure. Furthermore, the 953bp *ramAp* sequence, including its putative upstream promoter, was BLASTN-searched on NCBI to yield plasmids with 100% identity and coverage.

## 5. Conclusions

This study reports a concerning picture of *Salmonella* Paratyphi B in Jiangsu Province, China. For the first time, human infections linked to the XDR ST2814 and MDR ST42 strains were identified. Unraveling the resistance mechanisms revealed a complex interplay of mobile genetic elements like plasmids and ICEs, evolving resistance elements, and regulation of multidrug pumps in *Salmonella* Paratyphi B. This highlights the urgent need for heightened surveillance and improved control measures to curb this burgeoning public health threat.

## Figures and Tables

**Table 1:** List of primers for relative expression analysis of ramAp-related genes.

Gene	Primer	Primer Sequence (5′→3′)
*acrA*	*acrA*-F	CCTACCAGGCGACTTACGAC
	*acrA*-R	CGCCTGATCGTATTCCTGCT
*acrB*	*acrB*-F	TGCCCTGTATGCTATCTCGC
	*acrB*-R	ACCAGCATTACGGAGAACGG
*tolC*	*tolC*-F	GATCCTGCTCGTTCAGCGTA
	*tolC*-R	TTGACGTACTGGATGCCACC
*rpoB*	*rpoB*-F	GTTGAAAAAGGCCGTCGCAT
	*rpoB*-R	GCTCGCCAGTAGATTCGTCA

## Data Availability

The GenBank accession numbers for plasmids SA20019p_295K, TY20019p_286K, TY21021p_286K, TY_21030p_287K, are OR782950, PP024953, PP024954, PP024955, respectively; strain TY1512′s accession number is NZ_CP141594.1; the clean data accession number of the transconjugant ECSA22019 is SRR28415898.

## Supplementary Materials

The following supporting information can be downloaded at: https://www.mdpi.com/article/10.3390/antibiotics13060519/s1, Table S1: The AST results of strains in this study, Table S2: The information of relevant plasmids analyzed in this study.
